# Molecular basis for an attenuated mitochondrial adaptive plasticity in aged skeletal muscle

**DOI:** 10.18632/aging.100083

**Published:** 2009-09-12

**Authors:** Vladimir Ljubicic, Anna-Maria Joseph, Peter J. Adhihetty, Julianna H. Huang, Ayesha Saleem, Giulia Uguccioni, David A. Hood

**Affiliations:** ^1^ School of Kinesiology and Health Science, York University, Toronto, Ontario, M3J 1P3, Canada; ^2^ Muscle Health Research Centre, York University, Toronto, Ontario, M3J 1P3, Canada; ^3^Department of Biology, York University, Toronto, Ontario, M3J 1P3, Canada

**Keywords:** subsarcolemmal mitochondria, intermyofibrillar mitochondria, chronic contractile activity, performance, protein import, apoptosis

## Abstract

Our intent was to investigate the mechanisms driving the adaptive potential of subsarcolemmal (SS) and intermyofibrillar (IMF) mitochondria in young (6 mo) and senescent (36 mo) animals in response to a potent stimulus for organelle biogenesis. We employed chronic electrical stimulation (10 Hz, 3 h/day, 7 days) to induce contractile activity of skeletal muscle in 6 and 36 mo F344XBN rats. Subsequent to chronic activity, acute stimulation (1 Hz, 5 min) in situ revealed greater fatigue resistance in both age groups. However, the improvement in endurance was significantly greater in the young, compared to the old animals. Chronic muscle use also augmented SS and IMF mitochondrial volume to a greater extent in young muscle. The molecular basis for the diminished organelle expansion in aged muscle was due, in part, to the collective attenuation of the chronic stimulation-evoked increase in regulatory proteins involved in mediating mitochondrial protein import and biogenesis. Furthermore, adaptations in mitochondrial function were also blunted in old animals. However, chronic contractile activity evoked greater reductions in mitochondrially-mediated proapoptotic signaling in aged muscle. Thus, mitochondrial plasticity is retained in aged animals, however the magnitude of the changes are less compared to young animals due to attenuated molecular processes regulating organelle biogenesis.

## Introduction

Adult skeletal muscle is a highly malleable tissue which can respond positively to pharmacological, environmental, and mechanical stimuli with remarkable adaptations. A characteristic example of adaptive muscle plasticity is mitochondrial biogenesis. Increased organelle synthesis ultimately occurs as the result of the functional coordination between nuclear, cytosolic, as well as mitochondrial domains [1]. During the induction of organelle biogenesis, stress-sensitive signaling molecules, such as AMP-activated protein kinase (AMPK) and p38 mitogen-activated protein kinase (MAPK), communicate with downstream effectors including peroxisome proliferator-activated receptor γ co-activator 1α (PGC-1α) that results in the transcriptional upregulation of nuclear genes encoding mitochondrial proteins [2-4]. Newly-synthesized proteins which are destined for the organelle, such as mitochondrial DNA (mtDNA) transcription factor A (Tfam) and apoptosis-inducing factor (AIF), are directed to their specific mitochondrial sub-compartments by the mitochondrial protein import machinery (PIM). The PIM is comprised of translocase proteins of the outer mitochondrial membrane (TOM), as well as a similar complex within the inner membrane (TIM proteins). The coordination of these events, including the expression of mtDNA-encoded proteins by Tfam, leads to mitochondrial biogenesis. This results in morphological as well as functional alterations in the organelle, such as increased enzyme activity, respiratory capacity, and reticular expansion throughout the myofibers.

Although all mitochondria serve a similar function in providing ATP for the energy demands of the cell, electron microscopy has revealed regional differences in the subcellular location of muscle cell mitochondria [5,6]. Mitochondria that are clustered in proximity to the sarcolemma are termed subsarcolemmal (SS) mitochondria, and those embedded among the myofibrils are called intermyofibrillar (IMF) mitochondria. Biochemical investigations have shown that isolated IMF mitochondria contain lower levels of the phospholipid cardiolipin, but have higher enzyme activities, respiratory and protein synthesis rates, as well as elevated import rates of precursor proteins [7-11]. Furthermore, inherent differences in reactive oxygen species (ROS) production, as well as apoptotic and autophagic signaling have been previously documented [10,12]. In adults, the mitochondrial subfractions differ in their adaptability to a common stimulus, such as chronic muscle use [5,13,14], suggesting that their location within the cell makes them differentially sensitive to a common intracellular signal.

Skeletal muscle in aged animals is characterized by reductions in mass and the ability to develop force. This condition, known as sarcopenia, is defined by increased fatigability and the atrophy or loss of muscle fibers. Several mechanisms have been proposed to cause age-related muscle fibre atrophy, including endocrine-mediated signaling [15], diminished muscle progenitor cell activity [16], alterations in amino acid metabolism [17], as well as apoptotic myocellular decay. An increased incidence of apoptosis, as well as the expression of pro-apoptotic proteins and mitochondrially-mediated cell death signaling, have been reported in aged skeletal muscle [18-20]. Decrements in the oxidative capacity of aged skeletal muscle is associated with the impairment of mitochondrial function, such as reduced electron transport chain complex activity, ATP synthesis, and increased ROS production [18,21,22]. Furthermore, some controversy exists regarding the potential for adaptive plasticity of skeletal muscle in old, compared to young animals. For example, Skorjanc et al. [23] demonstrated an unaltered adaptability of skeletal muscle energy metabolism, including markers of glycolysis and mitochondrial function, to chronic low-frequency electrical stimulation-induced contractile activity in the aging rat. In contrast, other studies of chronic muscle use have shown a loss of adaptive plasticity, evidenced by a significantly slower rate of change in citrate synthase activity and fatigue resistance as a result of aging [24]. In addition, when assessing mitochondrial biogenesis, it is important to note whether the organelles have been identified as SS or IMF subfractions, since these respective mitochondria possess unique biochemical and functional properties which affect their inherent malleability [25]. Thus, the purposes of this study were to investigate the adaptive plasticity of skeletal muscle SS and IMF mitochondria in old (36 months), compared to young (6 months) animals in response to period of augmented organelle biogenesis. We employed chronic electrical stimulation to evoke contractile activity of skeletal muscle in an effort to induce an increase in mitochondrial volume. As observed with exercise training [26], increased muscle use in response to chronic stimulation, an established experimental model of endurance-type training, is a well-documented stimulus for eliciting mitochondrial adaptations in skeletal muscle [1]. We hypothesized that mitochondrial adaptive plasticity would be evoked in both young and old animals, but that the extent of organelle remodeling would be attenuated in the muscle of aged animals. Our results provide revealing insight into the reduced adaptive potential of aging skeletal muscle.

## Results

### Contractile activity-induced changes in skeletal muscle mass and contractile characteristics are similar between young and old animals

Similar to our previous reports [18,32], the skeletal muscle from the 36 mo old animals was sarcopenic, evidenced by a significantly reduced TA muscle mass, lower maximal force production, as well as slower rates of muscle contraction and relaxation (Table 1). Chronic stimulation had no effect on multiple aspects of contractile function, with the exception of the maximal force production per mg of TA weight (TET/TAW) and the maximal rate of force development (+dF/dt), which were both significantly reduced in young and old animals after chronic stimulation. The TET/TAW was decreased by chronic contractile activity by approximately 20% in both age groups, while the +dF/dt was reduced by 40-50% in the young and old animals.

**Table 1.** Skeletal muscle characteristics, contractile properties, and SS and IMF mitochondrial yield. Values are reported as means ± SE; n = number in parentheses. TAW, TA weight; BW, body weight; TW, maximum twitch force; TET, maximum tetanic force; TPT, time to peak twitch tension; 1/2 RT, half relaxation time; +dF/dt, rate of force development; -dF/dt, rate of relaxation; SS, subsarcolemmal; IMF, intermyofibrillar; Fold, fold difference; ¶ P < 0.05, CON vs. STIM; * P < 0.05, 6 mo vs. 36 mo.

				
	Muscle characteristics				Contractile properties				Protein yield	
	TAW (mg)	TAW/ BW (mg/g)	TW/ TAW (mN/mg)	TET/ TAW (mN/mg)	TPT (msec)	1/2 RT (msec)	+dF/dt (N/s)	-dF/dt (N/s)	SS (mg/g)	IMF (mg/g)
										
6 mo CON	826 ± 10 (8)	2.10 ± 0.07 (8)	2.26 ± 0.23 (8)	9.91 ± 0.49 (8)	25.7 ± 1.3 (8)	26.9 ± 2.5 (8)	103 ± 11.0 (7)	51.7 ± 5.5 (8)	2.06 ± 0.15 (21)	3.12 ± 0.17 (22)
										
6 mo STIM	826 ± 77 (8)	2.18 ± 0.12 (8)	1.96 ± 0.23 (8)	7.95^¶^ ± 0.49 (8)	25.0 ± 0.88 (8)	25.8 ± 4.4 (8)	62.2^¶^ ± 12.1 (6)	56.0 ± 7.8 (8)	2.56^¶^ ± 0.17 (22)	4.5^¶^ ± 0.19 (22)
Fold 6 mo STIM/CON	1.0	1.0	0.87	0.80	0.97	0.96	0.60	1.08	1.24	1.44
										
36 mo CON	498* ± 29 (8)	1.02* ± 0.05 (8)	2.75 ± 0.26 (7)	6.97* ± 0.88 (7)	28.4* ± 1.2 (6)	32.5* ± 1.39 (7)	48.1* ± 8.4 (5)	30.8* ± 1.3 (5)	2.71* ± 0.19 (17)	4.02* ± 0.32 (16)
										
36 mo STIM	474 ± 30 (8)	0.95 ± 0.04 (8)	2.35 ± 0.2 (8)	5.79^¶^ ± 0.49 (8)	27.9 ± 1.1 (8)	31.7 ± 1.74 (8)	25.4^¶^ ± 9.1 (6)	28.7 ± 2.1 (6)	2.6 ± 0.21 (17)	4.84^¶^ ± 0.36 (17)
Fold 36 mo STIM/CON	0.95	0.93	0.85	0.83	0.98	0.98	0.53	0.93	0.96	1.2
										
Fold 36 mo/6 mo	0.60	0.49	1.22	0.70	1.11	1.21	0.47	0.60	1.32	1.29
										

### Activity-induced improvements in muscle performance are attenuated in aged animals

**Figure 1.**
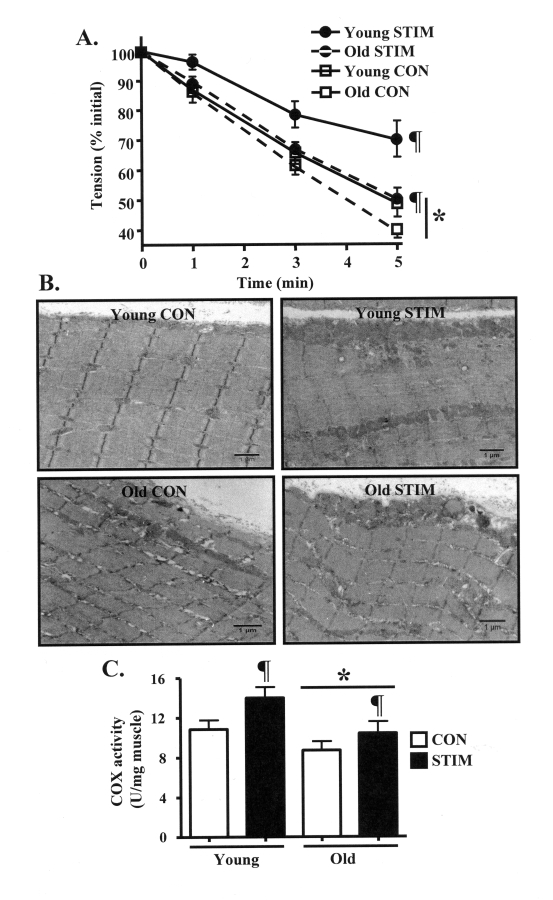
Chronic contractile activity-evoked increases in skeletal muscle endurance performance and mitochondrial content are reduced in old, compared to young animals. (A). Fatigue resistance during 5 min of 1 Hz *in situ* stimulation of the control (CON, open squares) and chronically stimulated (STIM, closed circles) tibialis anterior muscles from young (solid lines) and old (dashed lines) animals (*n *= 7-8). **(B) **Electron micrographs depicting skeletal muscle morphology and SS and IMF mitochondrial volumes in young and old, control (CON, open bars) and chronically stimulated (STIM, closed bars) extensor digitorum longus (EDL) muscle sections. All images were taken at the same magnification. Scale bar located at the lower right of each picture represents 1 μm. **(C) **COX enzyme activity in EDL muscle homogenates (*n *= 9-13). Data represent the mean ± SEM. * *P *< 0.05 vs. Young; ¶ *P *< 0.05 vs. CON.

Subsequent to 7 days of chronic contractile activity, we assessed the degree of fatigue resistance in the STIM and the CON limbs of both the young and old animals. After 5 min of acute, direct muscle stimulation, the force output of the TA muscle from the CON limb of the young animals declined to 49% of initial tension (Figure 1A). Skeletal muscle from the CON leg of old animals was significantly less fatigue resistant, as force was reduced to 39% of initial, representing a 20% difference between the age groups. In response to chronic contractile activity in the young animals, force was maintained at 69% of initial tension, which represented a 42% improvement (P < 0.05) compared to the CON limb. In the old animals, the decline in force output was also significantly attenuated, to 50% of initial tension. The chronic stimulation-induced 28% increase in fatigue resistance in the old animals was less than that observed in the young group. However, the muscle performance evident in the old animals after STIM resembled closely that documented in the CON limb of young animals.

### Chronic contractile activity augments muscle SS and IMF mitochondrial content to a greater extent in young animals

The physiologic assessments of skeletal muscle function were accompanied by biochemical and molecular assays of muscle and mitochondrial properties in young and old animals. Skeletal muscle SS and IMF mitochondrial volume was first qualitatively investigated using electron microscopy. In the CON muscle from young animals (Figure 1B, top left panel), the micrograph clearly shows a thick accumulation of SS mitochondria positioned beneath the sarcolemmal membrane, as well as the presence IMF mitochondria widely dispersed between the myofibrils. In contrast, a lesser volume of SS and IMF mitochondria was apparent in the CON limb from old animals (Figure 1B, bottom left). The adaptive response to chronic contractile activity included robust increases in organelle content in both the subsarcolemmal and intermyofibrillar regions of the muscle in both young and old animals (Figure 1B, bottom panels). Next, we quantitatively investigated muscle mitochondrial content by measuring cytochrome c oxidase (COX) activity, an established biochemical indicator of mitochondrial volume [1]. Similar to earlier reports [18], COX enzyme activity was 30% lower (P < 0.05) in the muscle from old, compared to young animals (Figure 1C). Chronic stimulation significantly elevated mitochondrial content in both young and old animals, however the increase was greater (30%; P < 0.05) in the muscle from young animals, compared to the 20% increase observed in the old animals. Furthermore, chronic contractile activity also increased the yield of SS and IMF mitochondria obtained during the mitochondrial isolation process to a greater extent in the young, compared to the old animals (Table 1).

### Chronic activity increases the expression of mitochondrial biogenesis regulatory proteins in the muscle from young and old animals

**Figure 2.**
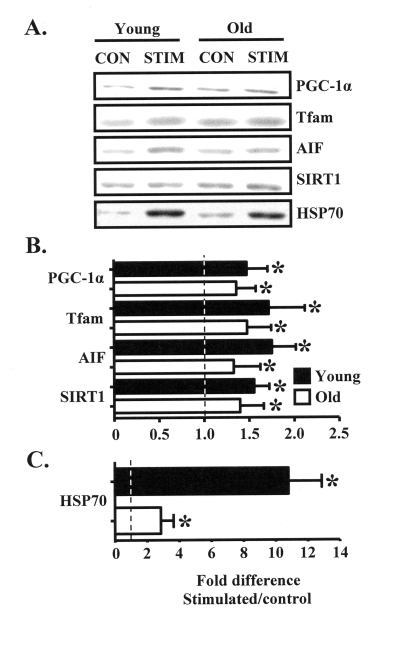
Chronic muscle use increases the expression of muscle and mitochondrial regulatory proteins in young and old animals. (A). Representative Western blots and graphical summary **(B) **of the effects of chronic contractile activity on the expression levels of proteins important for mitochondrial biogenesis (PGC-1α, Tfam), apoptotic signaling (AIF, HSP70), and aging (SIRT1), in muscles from young (closed bars) and old (open bars) animals expressed as the fold increase in chronically stimulated over control muscles (*n *= 6-11). **(C) **HSP70 protein content is shown separately due to the difference in scale, compared to the data in B. Data represent the mean ± SEM. * *P *< 0.05, stimulated vs. control.

In an effort to understand the molecular basis for the aging-associated attenuation of mitochondrial and muscle plasticity in response to chronic contractile activity, we employed Western blotting to measure the contents of 1) the critical mitochondrial biogenesis regulatory proteins PGC-1α and Tfam, 2) molecules important for mitochondrial and muscle function such as AIF and HSP70, as well as 3) SIRT1, a protein involved in the aging process. Chronic contractile activity significantly increased the expression of PGC-1α, Tfam, AIF, and SIRT1 in the muscle of young animals by approximately 50-65%, compared to the control limb (Figure 2A, B). Protein expression was also increased by 40-50% in the muscle of old animals, however the magnitude of this increase was lower compared to the young group. The stress protein HSP70 was highly induced in response to chronic stimulation, evidenced by the 3-fold and 11-fold increases in protein expression in the muscles from old and young animals, respectively (Figure 2C).

### SS and IMF mitochondrial protein import machinery components are increased after chronic stimulation in young, but not old animals

**Figure 3.**
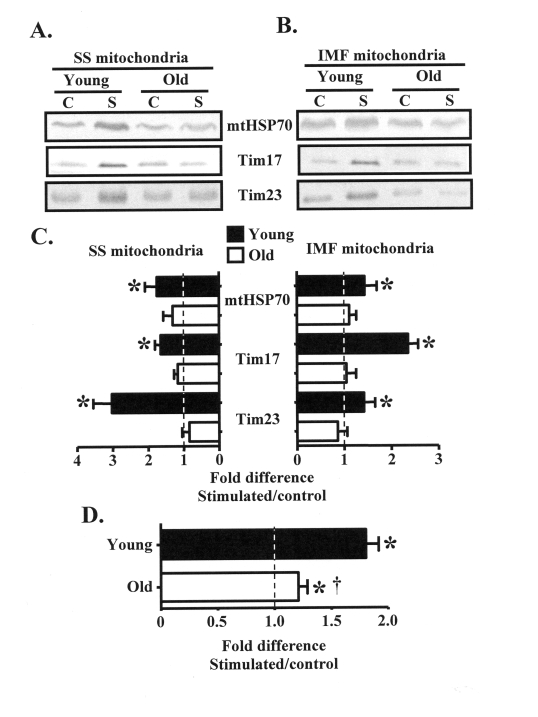
Subsarcolemmal (SS) and intermyofibrillar (IMF) mitochondrial protein import machinery components are increased in young, but not old animals in response to chronic contractile activity. (A). Representative Western blots of mtHSP70, Tim17, and Tim23 proteins in SS and IMF **(B) **mitochondrial subfractions isolated from the control (C) and chronically stimulated (S) limbs of young and old animals. **(C) **Graphical summary of the data in panels A and B expressed as the fold difference of the stimulated, over the control legs (*n *= 7-9). **(D) **Pooled results of the protein expression data in young, compared to old animals shown above in panel C, and panel B of Figure 3 (*n *= 74-86). Data represent the mean ± SEM. * *P *< 0.05, stimulated vs. control, **† ***P *< 0.05 vs. Young.

Expansion of the mitochondrial reticulum in response to organelle biogenesis-inducing stimuli requires the import of nuclear-encoded mitochondrial proteins. We therefore investigated contraction-evoked changes in the expression of mitochondrial protein import machinery, including mtHSP70, Tim17, and Tim23 in SS and IMF mitochondrial subfractions isolated from the control and chronically stimulated muscles of young and old animals. In the young group, chronic contractile activity augmented (P < 0.05) the expression of mtHSP70, Tim17, and Tim23 in SS mitochondria by approximately 2-3-fold, and in IMF mitochondria by 1.5-2-fold (Figure 3A-C). In contrast, the protein expression of these components of the import machinery did not increase within the mitochondria from old animals.

In an effort to summarize the effect of chronic contractile activity on the expression of proteins involved with mitochondrial plasticity, we pooled together results from Figures 2B and 3C. This analysis shows that chronic contractile activity significantly increased the expression of multiple proteins in the young animals, on average, by 1.8-fold above that found in the CON, non-stimulated muscle (Figure 3D). Chronic contractile activity also increased (P < 0.05) the expression of these proteins in the old animals by approximately 20% overall. However, the extent of the adaptation was attenuated (P < 0.05) in the old, compared to the young animals.

### Mitochondrial protein import is enhanced to a greater extent after chronic activity in muscle from young animals

**Figure 4.**
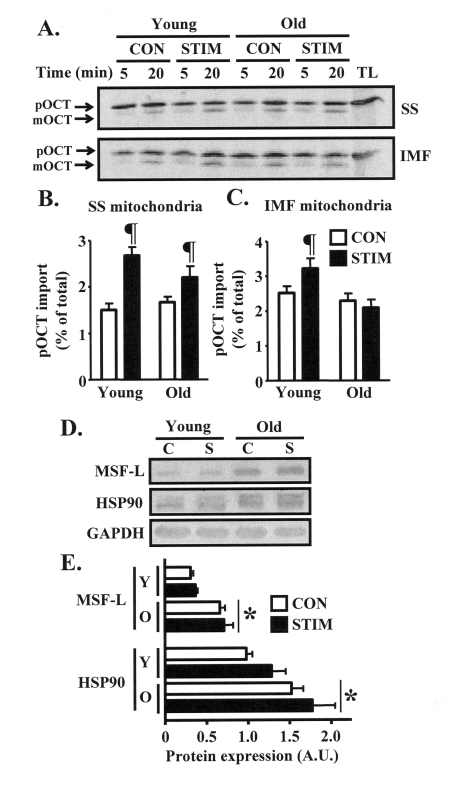
Mitochondrial import of the matrix protein ornithine carbamoyltransferase (OCT) is induced to a greater extent after chronic muscle use in young animals. (**A**) Representative autoradiograms of precursor (pOCT) and mature (mOCT) OCT after 5 and 20 min of the import reaction timecourse in isolated SS (top) and IMF (bottom) mitochondrial subfractions harvested from the control (CON, open bars) and chronically stimulated (STIM, closed bars) limbs of young and old animals (TL, translation lane without mitochondria). (**B**) and (**C**) Graphical summaries of the 20 min import data from repeated experiments shown in panel **A** (n = 9-12). (**D**) Western blots of MSF-L and HSP90 in isolated cytosolic fractions obtained from the control (**C**) and chronically stimulated (S) legs of young and old animals. GAPDH was used to confirm equal loading of protein. (**E**) Summary of repeated experiments shown in panel **D** (n = 5-7). Data represent the mean ± SEM. * P < 0.05 vs. Young; ¶ P < 0.05 vs. CON.

**Figure 5.**
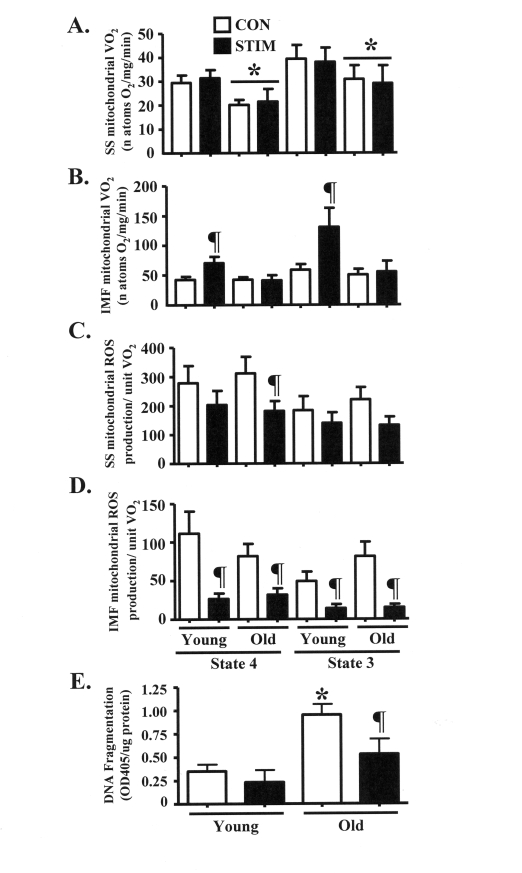
Chronic stimulation-induced adaptations in mitochondrial function and anti-apoptotic cell death signaling in young and old animals. (A). and **(B) **State 4 (2 μM rotenone and 10 mM succinate as substrates) and state 3 (rotenone and succinate plus 0.44 mM ADP) rates of oxygen consumption (VO_2_) in isolated subsarcolemmal (SS; **A**) and intermyofibrillar (IMF; **B**) mitochondria from the control (CON, open bars) and chronically stimulated (STIM, closed bars) limbs of young and old animals (*n *= 6-10). **(C) **and **(D) **State 4 and state 3 rates of reactive oxygen species (ROS) production per natom oxygen consumed in SS (**C**) and IMF (**D**) mitochondria from the CON and STIM limbs of young and old animals (*n *= 7-10). **(E) **Level of fragmented DNA, in the form of mono- and oligonucleosomes, in myonuclei-containing cytosolic extracts isolated from young and old animals (*n*: STIM = 4, CON = 21). Data represent the mean ± SEM. * *P *< 0.05 vs. Young; ¶ *P *< 0.05 vs. CON).

We next evaluated whether impaired adaptations in mitochondrial protein import machinery components in old animals would coincide with reduced functional rates of protein import into the organelle. Thus, we assessed the import of the matrix protein ornithine carbamoyltransferase (OCT) into isolated SS and IMF mitochondria harvested from the control and chronically stimulated muscles of young and old animals. Chronic stimulation significantly increased the import of OCT into the SS subfraction by 1.8-fold in the young animals, and 1.3-fold in the old group (Figs. 4A, B). Chronic contractile activity also resulted in a 30% induction (P < 0.05) in OCT import into the IMF mitochondria isolated from the young group, whereas there was no effect observed in the IMF subfraction from old animals (Figure 4A, C). In contrast, the content of protein chaperones MSF-L and HSP90, both involved in shuttling mitochondrial precursor proteins during cytosolic transit to the organelle, was not affected by chronic contractile activity (Figure 5D-E). However, the basal expression of these proteins was 40-100% higher (P < 0.05) in the skeletal muscle of old, compared to young animals.

### Succinate-stimulated mitochondrial oxygen consumption is increased in young, but not old animals after chronic contractile activity

 Previous assessments of glutamate-stimulated state 4 and 3 mitochondrial oxygen consumption (VO_2_) through complex I in our laboratory showed no difference between age groups [18]. Thus, organelle function was further assessed by measuring rates of complex II-driven VO_2_ in isolated SS and IMF mitochondrial subfractions. SS mitochondrial VO_2_ was 20-30% lower (P < 0.05) in old animals during both state 4 and state 3 VO_2_ driven in the presence of succinate (Figure 5A). Chronic stimulation not did alter SS mitochondrial VO_2_ in either age group. Rates of VO_2_ were significantly increased by 70-100% in IMF mitochondria from young animals in response to chronic contractile activity (Figure 5B). In contrast, the rate of VO_2_ of the IMF mitochondrial subfraction isolated from the old group was not affected by the treatment.

### Chronic muscle use evokes similar adaptations in mitochondrial reactive oxygen species (ROS) production in young and old animals

ROS production is an inherent metabolic byproduct of mitochondrial respiration within the organelle. Therefore, we measured succinate-stimulated ROS production and expressed the findings per unit of mitochondrial VO_2_. In young animals, chronic contractile activity did not influence the rate of ROS production from the SS mitochondrial subfraction (Figure 5C). However, in SS mitochondria isolated from old animals, state 4 ROS production was reduced by 40% after chronic stimulation (P < 0.05). In addition, state 3 ROS production tended to be lower (40%; 0.05 < P < 0.1) after chronic contractile activity. In the IMF mitochondria, chronic stimulation reduced ROS production by 60-80% (P < 0.05) in both the young and old groups (Figure 5D).

### Chronic contractile activity attenuates the elevated basal levels of myonuclear DNA fragmentation in the skeletal muscle of old animals

We investigated a downstream consequence of pro-apoptotic ROS signaling by assessing DNA fragmentation in cytosolic extracts isolated from young and old animals. The basal level of DNA fragmentation was approximately 3-fold greater (P < 0.05) in muscle from old, compared to young animals (Figure 5E). Chronic contractile activity had no influence on DNA fragmentation in young animals, however the level of fragmented DNA was significantly reduced by 45% in the old animals.

## Discussion

The intent of the present study was to examine the adaptive potential of skeletal muscle mitochondria in old animals in response to a potent stimulus for organelle expansion. To rapidly evoke SS and IMF mitochondrial biogenesis, we used chronic electrical stimulation-induced contractile activity of skeletal muscle, a well-established treatment to augment mitochondrial content [1,33]. This model allows for the elimination of any likely behavioural differences between young and old animals, and presents a standardized, high intensity contractile stimulus to the muscle. Our data illustrate that old animals retain the adaptive capacity for skeletal muscle and mitochondrial plasticity, however the extent of this remodeling was attenuated when compared to younger animals. Novel mechanistic insight for these findings is provided by the blunted contractile activity-induced elevations in mitochondrial biogenesis regulatory proteins, as well as the reduced potential for mitochondrial protein import. Notably however, molecular markers indicative of mitochondrially-mediated cell death signaling displayed similar, or greater improvements in the muscle from aged, compared to young animals in response to chronic contractile activity.

Skeletal muscle from healthy adult animals is highly responsive to stimuli such as chronic contractile activity [1,34]. In an effort to further our understanding of the aging-associated alterations in skeletal muscle biology, we compared young adult to senescent animals, which present with a high degree of sarcopenia. Indeed, aging-evoked muscle pathology was evidenced by a 40-50% lower muscle mass, as well as significant reductions in maximal force-producing capacity and slower rates of contraction. While chronic contractile activity induced only modest changes in the skeletal muscle contractile properties of young and old animals, which were similar between the age groups, this treatment resulted in significant adaptations in muscle fatigue resistance. Moreover, chronic contractile activity effectively rescued the aging-induced decline in muscle performance, resulting in a younger phenotype in the old animals. However, the magnitude of the increase was greater in the young, compared to the old group, indicative of an attenuated adaptive plasticity of pathways involved in oxidative metabolism in aging muscle. This was certainly related in large measure to the greater increase in overall mitochondrial content in young animals, which was confirmed by assessments of multiple indices of mitochondrial volume, including COX enzyme activity, as well as electron microscopy and yield of SS and IMF mitochondrial subfractions. It is well known that the content of mitochondria is closely correlated with muscular endurance performance [35]. Thus, chronic stimulation evoked adaptive plasticity in the performance of aging muscle, which was based, in part, on increased SS and IMF mitochondrial volume. The greater increase in organelle biogenesis observed in young animals suggests that the molecular mechanisms driving mitochondrial synthesis in response to chronic muscle use are less responsive in old animals.

During the process of mitochondrial biogenesis, a number of proteins have been demonstrated to play important roles in the proper assembly and function on the organelle. These factors include the critical nuclear and mitochondrial genome transcriptional regulatory proteins PGC-1α and Tfam, as well as the anti-apoptotic stress molecule HSP70, and the mitochondria-localized AIF [25,36,37]. Collectively, the content of these proteins were augmented in aging muscle in response to chronic contractile activity, however the increase was lower than that observed in the younger animals. We conclude that the reduced plasticity of mitochondria in aged muscle is partly due to the blunted expression of these factors in response to chronic contractile activity. This phenomenon is likely the result of diminished upstream contraction-induced signaling to mitochondrial biogenesis, as recently described in aged, compared to young animals [32]. It has been previously shown that decreased levels of PGC-1α and Tfam depress mitochondrial biogenesis [38,39]. Further, AIF is a critical component for the maintenance of normal mitochondrial cristae structure and oxidative phosphorylation [37,40]. Mice deficient in AIF exhibit fragmented organelles of punctuate morphology [37]. While there are conflicting reports regarding the role of the SIRT1 longevity factor in skeletal muscle mitochondrial biogenesis [41-43], as well as its expression in response to chronic muscle use  [41,44], our data show, for the first time, that SIRT1 content is increased in both young and senescent animals coincident with the chronic contractile activity-evoked upregulation of mitochondrial content. This finding suggests that chronic muscle use may represent an effective component of a treatment regimen for aging-associated pathology, in part through enhanced SIRT1 expression, given its putative pro-survival function [45,46].

Post-transcriptional and -translational processing of nuclear-encoded mitochondrial gene products are essential for mitochondrial adaptations. The majority of mitochondrial proteins are encoded in the nucleus, and must be targeted and translocated to the mitochondrial subcompartment. The PIM, consisting of the TOM and TIM assembly complexes, is responsible for ushering these proteins and assembling them into a functional organelle. The components of this pathway and the mechanisms regulating this process remain poorly understood in skeletal muscle. Our previous work has demonstrated that specific PIM components, including HSP60, CPN10, Tom20, and Tom34 are highly inducible by chronic muscle use in adult muscle [29,47,48]. Data from the present study show that the expression of Tim23, Tim17, and mtHSP70 are induced with chronic stimulation in SS and IMF mitochondria isolated from the muscle of young, but not old animals. Thus, the diminished plasticity of mitochondria from aged muscle is associated with a collective attenuation in the adaptive response of proteins critical for organelle remodeling.

The PIM constituents that were examined here are responsible for targeting proteins that are destined for the mitochondrial inner membrane, intermembrane space, and matrix. We have previously demonstrated that the import rate of matrix-localized molecules, including Tfam and MDH, is increased during conditions of chronic contractile activity-induced mitochondrial biogenesis [29,49]. Our results support these earlier findings, as import of the matrix protein OCT was augmented in response to chronic muscle use in adult animals. In contrast, in aged muscle contractile activity did not affect OCT import into IMF mitochondria, while the magnitude of the increase in the SS subfraction was significantly lower, compared to the increase observed in the young animals. In the absence of any change in protein import machinery components, including auxiliary factors such as the cytosolic chaperones MSF-L and HSP90, the modest increase in OCT import into SS mitochondria from aged muscle may be attributed to potential alterations in other PIM components, such as HSP60, CPN10, Tim50, or Tim21 [50]. Thus, it seems reasonable to suggest that the attenuated protein import response in aged, compared to young muscle, as well as the muted adaptive plasticity of multiple protein factors involved in organelle synthesis, including the PIM components, reveals a mechanistic basis for the reduced level of mitochondrial biogenesis and muscle performance documented in old animals. Assessments of the insertion of discrete proteins into other mitochondrial compartments, such as the inner and outer membranes, as well as the assembly of multi-subunit enzyme complexes (e.g. COX, TOM), remain fertile areas of future investigations into the plasticity of muscle biological chemistry.

The decrement in the adaptive potential of aged muscle was also manifest by the functional evaluation of SS and IMF mitochondrial respiration in the presence of succinate. Whereas both state 4 and state 3 respiration were significantly elevated in the IMF subfraction from young animals, mitochondria from muscle of old animals did not adapt to chronic contractile activity. Farrar et al. [51] have previously shown that state 3 mitochondrial respiration was increased in SS and IMF subfractions isolated from young and old animals after a period of chronic muscle use. Notably, the training-induced increase in mitochondrial respiration was similar, or greater in the organelles isolated from the aged muscle. However, the authors employed a regimen of exercise training to evoke mitochondrial adaptations in animals that were only ~24 months of age. This represents a considerable difference in experimental design compared to the present study. These data suggest that the reduced adaptive plasticity of muscle in this model of organismal aging occurs between 24 and 36 months of age.

Excessive ROS production within the mitochondria acts as an early signal to initiate the mitochondrially-mediated cell death pathway, leading ultimately to myonuclear decay and apoptosis [52]. In adult animals, chronic muscle use reduces apoptogenic mitochondrial signaling in skeletal muscle, while muscle disuse has the opposite effect [14,53,54]. Our data illustrate that in IMF mitochondria, complex II-driven ROS production was decreased to a similar extent in organelles isolated from chronically stimulated young and aged muscle. This adaptation represents a significant reduction in pro-apoptotic signaling throughout the myofiber, in light of the fact that the IMF subfraction accounts for approximately 80% of the total mitochondrial volume in the cell [5]. Indeed, when we assessed the level of DNA fragmentation, the terminal step and hallmark indicator of apoptosis, we found that chronic contractile activity exerted a more powerful influence in reducing DNA fragmentation in aged, compared to young muscle. This adaptive response may be related to potential chronic stimulation-induced alterations in antioxidant and/or antiapoptotic signaling in the mitochondrial, cytoplasmic, or nuclear domains of aged muscle. Our data demonstrate a chronic stimulation-evoked increase in the antiapoptotic stress protein HSP70 in aged animals, and it is known that the apoptosis repressor with a caspase recruitment domain is also inducible in skeletal muscle in response to chronic contractile activity [14]. It is evident that skeletal muscle from older animals is more receptive to reductions in DNA catabolism which may be due, in part, to the high level of DNA fragmentation already apparent under basal conditions. We have shown previously that the muscle of young animals possesses a resistance to alterations in DNA fragmentation even under conditions of aggressive proapoptotic signaling evoked by chronic muscle disuse (i.e. denervation; [53]). Thus, chronic contractile activity elicits a robust antiapoptotic adaptive response in aged muscle, and suggests a heightened molecular plasticity in defense of the myonuclear decay and myofiber loss associated with the sarcopenia of aging.

In summary, the present study demonstrates that the adaptive plasticity of skeletal muscle and mitochondria is attenuated in aged, compared to young animals under conditions of chronic contractile activity-induced organelle biogenesis. Our data reveal novel insight into the molecular processes that are in part responsible for this decrement, including lesser elevations in important mitochondrial biogenesis regulatory factors, reduced signaling kinase activation [32], as well as decreased functional rates of SS and IMF mitochondrial protein import and ATP provision [32]. Despite this attenuated response, chronic contractile activity resulted in beneficial functional adaptations in a number of muscle and mitochondrial parameters in aged animals. This finding has obvious relevance for the development of potential pharmacological and/or lifestyle therapeutics, such as chronic physical activity, for aging-associated diseases including sarcopenia and diabetes.

## Methods

Animals. Experiments were conducted after approval by the York University Animal Care Committee in accordance with Canadian Council of Animal Care guidelines. Male Fischer 344 Brown Norway rats were obtained from the National Institute of Aging (Bethesda, MD) and divided into 6 mo (young) and 36 mo (senescent) groups. Animals were housed individually and given food and water ad libitum.

Chronic contractile activity. The procedure as outlined previously [9] was followed for implantation of electrodes and chronic low-frequency electrical stimulation of animals. Briefly, rats were anaesthetized, and under aseptic conditions, an internal stimulation unit encased in silicone [27] was secured to the interior of the abdominal musculature in the intraperitoneal cavity. Platinum electrode wires were passed subcutaneously and two stimulating electrodes were sutured unilaterally flanking the common peroneal nerve of the left hindlimb. Stimulation was adjusted at the time of electrode implantation to result in palpable contractions of the tibialis anterior (TA) and extensor digitorum longus (EDL) muscles. After a 1-week recovery period, the TA and EDL muscles were chronically stimulated (STIM; 10 Hz, 0.1 ms duration) 3 h/day for 7 days. The contralateral limb was used as a non-stimulated internal control (CON) in all animals. After the stimulation period, animals were anaesthetized and the in situ stimulation protocol was performed.

In situ acute stimulation . Approximately 21 hours after the last bout of chronic stimulation, the animals were anesthetized, and the chronically stimulated, as well as the contralateral control TA muscles from young and old animals were exposed and prepared for *in situ* direct muscle stimulation, as detailed earlier [28]. The distal tendon of each TA muscle was isolated, and a hooked pin was affixed to the tendon. The pin of one limb was attached to a strain gauge, while the other leg was misted with saline and wrapped in plastic to prevent dehydration. Intramuscular stimulating electrodes were placed in the belly of the muscle, parallel to the fibers. The experimental protocol involved stimulation with 100 ms trains at 100 Hz to determine maximal tetanic tension produced by the muscle. This was followed by a stimulation period of 5 min at a frequency of 1 Hz (0.1 ms duration) to evaluate muscle performance during fatigue-inducing conditions. Force and pressure signals were sampled online (Powerlab 4/SP, ADInstruments, Colorado Springs, CO) and stored for analysis using Chart 5 software. Immediately upon the cessation of contractions, the TA muscle of the acutely stimulated limb was quickly harvested, weighed, and placed in ice-cold mitochondrial isolation buffer 1. The EDL muscle was sectioned, with one portion freeze-clamped with aluminum tongs pre-cooled in liquid nitrogen, and stored at -70 °C for use in subsequent and cytochrome c oxidase (COX) enzyme activity measurements and Western blotting analyses, while the other portion was prepared for serial sectioning and electron microscopy. Acute stimulation and sampling of the TA and EDL muscles from the contralateral limb followed. Animals were then sacrificed by exsanguination after a medial thoractomy.

Isolation of mitochondrial and cytosolic fractions. The TA muscles were briefly minced, and the SS and IMF mitochondria were fractionated by mechanical disruption, differential centrifugation, and 0.025 ml/g tissue protease digestion as described previously in detail [10]. Cytosolic extracts were prepared concurrently during this process as outlined earlier [29]. Mitochondria were resuspended (100 mM KCl, 10 mM MOPS, 0.2% BSA) and an aliquot of the suspension was taken for measurements of protein content [30], and the yield was expressed as mg/g muscle wet weight.

Mitochondrial respiration. Samples of isolated SS and IMF mitochondrial subfractions were incubated with 250 μl of VO_2_ buffer (250 mM sucrose, 50 mM KCl, 25 mM Tris-HCl, and 10 mM K_2_HPO_4_, pH 7.4) at 30 °C in a water-jacketed respiratory chamber with continuous stirring. Respiration rates (n atoms O_2_•min^-1^•mg^-1^) driven by complex II in the mitochondrial electron transport chain were evaluated in the presence of 2 μM rotenone and 10 mM succinate (state 4 respiration), or rotenone and succinate plus 0.44 mM ADP (state 3 respiration) using the Mitocell S200 Micro Respirometry System (Strathkelvin Instruments, Motherwell, UK). The addition of NADH during state 3 measurements had no effect on the respiration rate (data not shown), indicating excellent mitochondrial membrane integrity.

Mitochondrial ROS production . ROS were measured as described previously [14]. Briefly, SS and IMF mitochondria (50 μg) from young and old animals were incubated with VO_2_ buffer in a 96-well plate. ROS production was assessed at 37 °C for 30 min during state 4 and state 3 respiration by adding 2 μM rotenone and 10 mM succinate, or rotenone and succinate plus 0.44 mM ADP, respectively, immediately prior to the addition of 50 μM dichlorodihydrofluorescein diacetate. The fluorescence emission between 480-520 nm measured with a multi-detection micro-plate reader (Synergy HT, Biotek Instruments Inc., Winooski, VT) is directly related to ROS production. Data were recorded and interpreted using KC4 (v 3.0) software. ROS production measured in absolute fluorescence units was linear over the entire measurement period. ROS levels were expressed per natom of O_2_ consumed, measured during the mitochondrial respiration assay.

DNA isolation and *in vitro* transcription. The plasmid containing the full-length cDNA encoding precursor ornithine carbamoyltransferase (pOCT) was isolated from bacteria using an alkaline lysis method. The cDNA resulting from this preparation was linearized with Sac I at 37°C for 2 hours. Plasmid DNA was extracted with phenol and precipitated in ethanol overnight at -80°C. DNA, at a final concentration of 0.8 μg/μl, was transcribed with SP6 RNA polymerase, ribonucleoside triphosphate substrates and the cap analog m7G(5')ppp(5')G at 40°C for 90 min. The pOCT mRNA was extracted with phenol and precipitated in ethanol at -80°C overnight. mRNA was resuspended in sterile distilled water and adjusted to a final concentration of 2.8 μg/μl. Aliquots were stored at -20°C for *in vitro* translation assays.

*In vitro* translation and mitochondrial protein import. The pOCT mRNA was translated and labeled with the use of a rabbit reticulocyte lysate system in the presence of [^35^S]-methionine. Freshly isolated SS and IMF mitochondria and the translated radiolabeled precursor proteins were equilibrated separately at 30°C for 10 min. The translated precursor proteins were added to the mitochondrial samples and incubated at 30°C to initiate the protein import reaction. Equal aliquots of the import reaction were withdrawn at 0, 5, and 20 min to determine basal pOCT import rates in control and chronically active muscle from young and aged animals. Final import reactions consisted of 25 μg of mitochondria and 12 μl of the lysate containing the radiolabeled precursor protein. Mitochondria were then recovered by centrifugation through a 20% sucrose cushion for 15 min at 4°C. Pellets were resuspended, lysed and then separated using 8% SDS-PAGE. After electrophoresis, gels were boiled for 5 min in 5% TCA, rinsed for 30 seconds in distilled water, followed by rinsing in 10 mM TRIS (5 min) and 1 M sodium salicylate (30 min). Gels were subsequently dried for ~ 1 hour at 80°C and exposed overnight to a Kodak Phosphor screen. Total intensities were quantified (Quantity One, Bio-Rad). Import was expressed as the percent of processed mature protein (mOCT) per minute, relative to the total protein available.

DNA fragmentation. Aliquots of cytosolic extracts [29] from young and old animals were prepared for spectrophotometric detection of DNA fragments, in the form of mono- and oligonucleosomes, as per the manufacturers instructions (Cell Death Detection ELISA^PLUS^, Roche Applied Science, Laval, PQ).

Electron microscopy. EDL muscles from the CON and STIM legs of young and old animals were excised and cut at mid-belly to obtain 2-3 mm serial sections. Muscle samples were incubated on ice for 1 hour in 3.0% glutaraldehyde buffered with 0.1 M sodium cacodylate. Sections were then washed three times in 0.1 M sodium cacodylate buffer before being post-fixed for 1 hour in 1% osmium tetroxide in 0.1 M sodium cacodylate at room temperature. Muscle sections were then dehydrated by washes with 30%, 50%, 80% and 100% ethanol, then in ethanol-propylene oxide for 1 hour, and followed by 100% propylene oxide for 1 hour. Subsequently, muscle sections were left overnight in a propylene oxide-epon resin mixture in a glass dessicator. Groups of muscle fibers were then dissected from the sections, embedded in fresh resin and incubated at 60°C for 48 hours. Ultrathin sections (60 nm) were cut, collected on copper grids, and stained with uranyl acetate and lead citrate. Electron micrographs were obtained using a Philips EM201 electron microscope.

Cytochrome c oxidase (COX) enzyme activity. COX activity of the EDL muscles from CON and STIM limbs was evaluated as described previously [31]. Enzyme activity was determined spectrophotometrically at 30 °C as the maximal rate of oxidation of fully reduced cytochrome c, measured by the change in absorbance at 550 nm.

Western blotting. Frozen EDL sections from CON and STIM limbs of young and old animals were pulverized to a fine powder with a stainless steel mortar that was cooled to the temperature of liquid nitrogen. The protein extraction was performed as previously described [31]. Proteins extracted from the muscle homogenates, isolated mitochondria, or cytosolic samples were resolved by SDS-PAGE (10-12% polyacrylamide) and subsequently electroblotted to nitrocellulose membranes (Amersham, Baie D'Urfé, PQ). After transfer, membranes were blocked (1 h) with a 5% skim milk in 1 X TBST [Tris-buffered saline-Tween 20: 25 mM Tris•HCl (pH 7.5), 1 mM NaCl, and 0.1% Tween 20] solution. Blots were then incubated in blocking solution with antibody directed against PGC-1α (Calbiochem, 516-557), Tfam, apoptosis-inducing factor (AIF; Santa Cruz, sc-9416), sirtuin 1 (SIRT1; Sigma, S5313), heat shock protein 70 (HSP70; Stressgen, SPA-810), mitochondrial HSP70 (mtHSP70; Stressgen, SPS-825), the translocase of the inner mitochondrial membrane 17 (Tim17; Santa Cruz, sc-13293), Tim23 (BD Bioscience, 611222), mitochondrial import-stimulating factor (MSF-L; gifted by Dr. K. Mihara, Kyushu University), HSP90 (Stressgen, SPA-845), and glyceraldehyde-3 phosphate dehydrogenase (GAPDH; Abcam, ab8245) overnight at 4 °C. After 3 X 5 min washes with TBST, blots were incubated at room temperature (1 h) with the appropriate secondary antibody coupled to horseradish peroxidase. Blots were then washed again 3 X 5 min with TBST, followed by visualization with enhanced chemiluminescence. Films (Hyperfilm, Amersham) were then scanned and analyzed using SigmaScan Pro 5 software (Jandel Scientific, San Rafael, CA).

Statistics. The data were analyzed using paired and unpaired Student's t-tests and analysis of variance (ANOVA) procedures, as appropriate. Bonferroni's post hoc test was used to test significant differences revealed by the ANOVA. Statistically significant distinctions between groups represented in the graphs depicted as fold differences are computed using the raw data sets prior to conversion to the fold difference values. Significance was accepted at P < 0.05.
